# COVID-19 in a Pediatric Patient: Novel Presentation of Cardiac Failure resulting in Chylous Ascites and Abdominal Pain

**DOI:** 10.1097/PG9.0000000000000068

**Published:** 2021-03-30

**Authors:** Claudia Phen, Joseph Woolley, Shannon Kelley, Jessica Garcia, Nathanya Baez Hernandez, Amal Aqul

**Affiliations:** From the * Division of Pediatric Gastroenterology, Hepatology and Nutrition, Department of Pediatrics, University of Texas Southwestern/Children’s Medical Center, Dallas, TX; † Division of Pathology, Department of Pediatrics, University of Texas Southwestern/Children’s Medical Center, Dallas, TX; ‡ Division of Hematology, Department of Pediatrics, University of Texas Southwestern/Children’s Medical Center, Dallas, TX; § Division of Cardiology, Department of Pediatrics, University of Texas Southwestern/Children’s Medical Center, Dallas, TX.

## INTRODUCTION

The novel severe acute respiratory syndrome coronavirus 2 (SARS-CoV-2) resulting in coronavirus disease 2019 (COVID-19) continues to cause a worldwide pandemic. Recognized clinical presentations of COVID-19 have evolved beyond solely respiratory symptoms. We report a 13-year-old girl with severe abdominal pain who was found to have chylous ascites, cardiac dysfunction, and multiple thrombi 10 weeks following a COVID-19 infection. Informed consent was obtained from the family before the submission of this article.

## CASE REPORT

A 13-year-old girl with a history of Ewing Sarcoma diagnosed in 2009, post left leg amputation and rotationplasty in remission since 2010 acutely developed nausea, nonbilious, nonbloody emesis, and severe cramping right lower quadrant abdominal pain, prompting admission for evaluation. Ten weeks before admission, she had tested positive for SARS-CoV-2 on real-time reverse transcription-polymerase chain reaction via nasopharyngeal swab when she had presented with fever, fatigue, and cough.

Laboratory findings on admission were remarkable for leukocytosis, thrombocytosis, elevated inflammatory marker, low albumin, elevated aminotransferases, and prolonged prothrombin time (Table 1). Electrolytes were normal. Computed tomography (CT) abdomen revealed moderate free abdominal fluid and right-sided pleural effusion. Liver ultrasound with Doppler showed mild hepatomegaly, thickened gallbladder wall, normal spleen, and patent hepatic artery, hepatic and portal veins, and inferior vena cava.

**TABLE 1.** Laboratory results at admission and during hospitalization

Laboratory measures	HD 0	HD 5	HD 16	Reference range
White blood cell count, 1000/mm^3^	14.6	12.8	11.2	4.5–11.0
Lymphocytes, %	24.9	31.0	30.9	24.0–44.0
Neutrophils, %	63.1	62.1	54.2	36.0–66.0
Hemoglobin, g/dL	12.2	11.7	11.0	12.0–15.0
Platelet count, 1000/mm^3^	572	279	678	150–450
Albumin, g/dL	3.1	2.9	3.3	3.6–5.0
Triglycerides, mg/dL	112			
Prothrombin time, s	20.5	18.1	13.5	12.0–15.0
International normalized ratio	1.7	1.5	1.0	
Total bilirubin, mg/dL	1.6	0.6	0.3	0.1–1.3
Direct bilirubin, mg/dL	0.7	0.4	0.1	0.1–0.3
Alanine aminotransferase, U/L	174	531	37	10–50
Aspartate aminotransferase, U/L	176	327	15	10–45
C-reactive protein, mg/dL	2.3	6.9	0.7	0–1.0
Lipase, units/L	24			31–93
D-dimer, mcg/mL	10.0	8.64	1.50	0–0.5
ESR, mm/h	3.0	16.0	44.0	0–20.0
Troponin, ng/mL		0.016	1.060	<0.100
BNP, pg/mL		1437.7	1316.30	<100.0

BNP = B-type natriuretic peptide; ESR = erythrocyte sedimentation rate; HD = hospital day.

**FIGURE 1.**
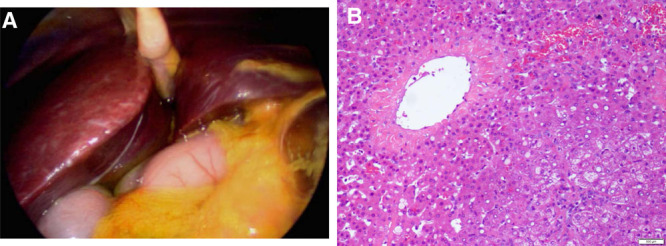
Gross and histopathologic findings of the liver at time of diagnostic laparoscopy. A) Patchy, inflamed appearance of the liver with a large amount of thick, yellow ascites visualized in the upper abdomen on diagnostic laparoscopy. B) Biopsy of the liver showing centrilobular (zone 3) sinusoidal dilatation and congestion with rare zone 3 hepatocyte dropout. Periportal (zone 1) hepatocytes show feathery degeneration and balloon change.

Due to the severity of abdominal pain, ascites, and history of Ewing sarcoma, she underwent diagnostic laparoscopy, which revealed a large amount of ascites and patchy inflamed and ischemic appearance of the liver (Fig. 1A). The remainder of the abdominal inspection was unremarkable, and a peritoneal drain was placed. The initial ascitic fluid analysis revealed triglycerides (TGs) 75 mg/dL with normal bilirubin. Cytopathology of the peritoneal fluid showed predominantly foamy, nonpigmented macrophages. No infectious organisms or malignant cells were visualized, and cultures were negative. Liver biopsy demonstrated central lobular congestion suggestive of veno-occlusive disease and vascular compromise without signs of malignancy or inflammation (Fig. 1B). On hospital day (HD) 2, her peritoneal fluid became milky in appearance with elevated TG (219 mg/dL).

**FIGURE 2.**
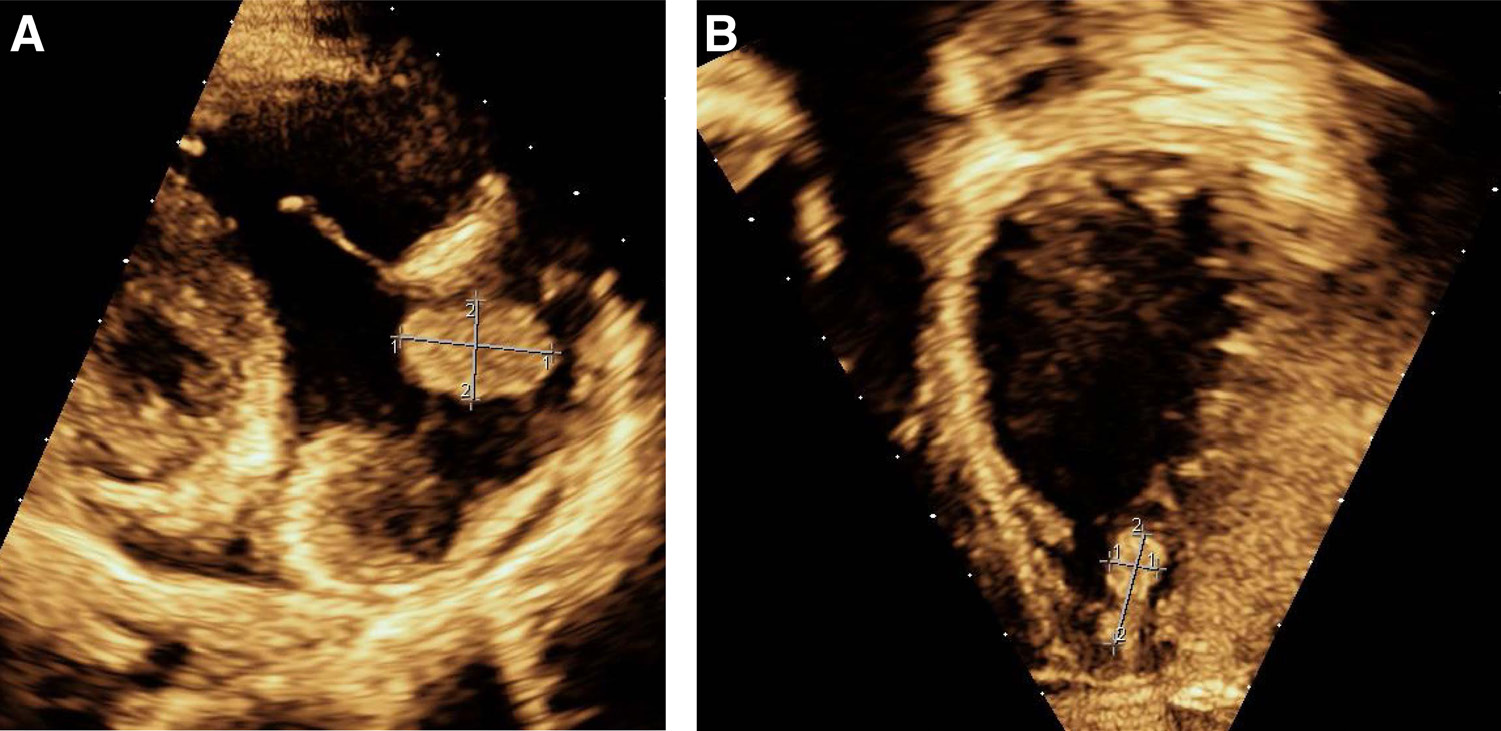
Echocardiogram demonstrating (A) an echogenic mass (2.3 × 1.5 cm) in the right atrium and (B) another echogenic mass (0.7 × 1.6 cm) in the left ventricle.

Additional tests for congestive hepatopathy revealed elevated B-type natriuretic peptide (1437 pg/mL) and an abnormal echocardiogram with severely depressed systolic function (ejection fraction [EF] 28%) and large thrombi in the right atrium and left ventricle (Fig. 2). The patient had a history of doxorubicin exposure; however, her last surveillance echocardiogram 3 months before demonstrated normal biventricular systolic function. Hematology consults led to findings of elevated D-dimer levels and factor VIII 241% (normal: 53%–131%). Inherited (factor V Leiden and prothrombin G20210A gene mutation, protein C and S activity, and antithrombin III activity) and acquired thrombophilia (lupus anticoagulant screen, anticardiolipin antibodies, and anti-B2 glycoprotein) were negative. Ultrasound of upper and lower extremities showed no clots. She was admitted to the cardiovascular intensive care unit on milrinone and heparin drip. On HD 9, cardiac MRI revealed myocardial scarring in the anterior segments of the subendocardium characteristic of an ischemic cause. Lobar pulmonary emboli with two pulmonary infarcts were also visualized bilaterally on CT angiography. She was transitioned to aspirin and enoxaparin before discharge. Repeat echocardiogram obtained on HD 16 revealed improvement in EF (38%). She was discharged on carvedilol, digoxin, enalapril, and furosemide. On HD 10, the patient’s SARS CoV-2 IgG returned elevated at 5.07.

At a 6-month follow-up, she was clinically well without cardiac symptoms or abdominal pain. However, her systolic function remained depressed (~30%). Liver enzymes and bilirubin had normalized. CT heart demonstrated resolution of the intracardiac and pulmonary thrombi.

## DISCUSSION

The COVID-19 pandemic continues to cause catastrophic disease in the USA. Increasing cases of COVID-19 have been documented in children and adolescents. The presence of gastrointestinal symptoms in children with COVID-19 has been well described (1). Abnormalities in liver chemistries have been reported in 20%–30% of cases with COVID-19 (2). This is in contrast to adults who may present primarily with respiratory symptoms and infrequently with gastrointestinal involvement. The symptoms of nausea, vomiting, abdominal pain, or diarrhea can be mistaken for more common etiologies including gastrointestinal infections or inflammatory bowel disease.

Multisystem inflammatory syndrome in children (MIS-C) has been described with associated fever, elevated inflammatory markers, and multi-organ involvement (3,4). However, our patient did not fit the case definition for MIS-C based on Centers for Disease Control criteria and had a prolonged time passage from COVID-19 infection. It is not uncommon for patients with COVID-19 infection to have multi-organ dysfunction in the acute setting. In a multi-center series of 186 pediatric patients infected with SARS-CoV-2, 92% had gastrointestinal involvement, 80% had cardiovascular involvement, and 76% had hematologic involvement (3). Reports have also outlined greater thrombotic risk for patients with COVID-19 such as venous thromboembolism, pulmonary thrombosis, and myocardial infarction (5–7). However, few patients present with this degree of severe multi-organ disease weeks following primary COVID-19 infection.

The rapid progression to decompensated heart failure following our patient’s COVID-19 infection raises suspicion for viral myocarditis as a possible etiology. Her initial presenting symptom of abdominal pain was ultimately attributed to ischemia caused by systolic heart failure. Congestive heart failure has been reported to lead to the development of chylous ascites and may have been the trigger in our patient’s case (8–10). Heart failure leads to elevated central venous pressures which can increase capillary filtration and induce abdominal lymph production, increased peritoneal lymphatic pressure, and leakage of lipid-rich lymph into the peritoneal cavity (11). Although the level of TGs in our patient’s initial ascitic fluid does not meet diagnostic criteria (TG content >200 mg/dL), it is important to note that our patient had poor oral intake and malnutrition in the weeks from her COVID-19 test to the presentation. This may have led to a falsely low or normal TG content in her ascitic fluid.

This is the first reported case of systolic heart failure leading to severe abdominal pain and chylous ascites in the setting of prior SARS-CoV-2 infection. As we continue to witness the spread of COVID-19, it is important to think broadly about our differential diagnosis for a patient presenting with primarily gastrointestinal symptoms. Our patient’s case emphasizes the need to consider multisystem involvement, especially in pediatric patients with a history of SARS-CoV-2 infection.
